# Cephalomedullary Nailing vs. Dynamic Hip Screw for the Treatment of Pertrochanteric Fractures: The Role of Cytokines in the Prediction of Surgical Invasiveness

**DOI:** 10.3390/jcm14061825

**Published:** 2025-03-08

**Authors:** Armando Del Prete, Pasquale Sessa, Ferdinando Del Prete, Christian Carulli, Giacomo Sani, Mariangela Manfredi, Roberto Civinini

**Affiliations:** 1 Azienda Ospedaliero-Universitaria Careggi, Orthopedic Clinic, University of Florence, Largo Palagi, 1, 50139 Florence, Italy; armandodelprete@alice.it (A.D.P.); christian.carulli@careggi.it (C.C.); roberto.civinini@unifi.it (R.C.); 2 Department of Orthopedics and Traumatology, Azienda Ospedaliera San Camillo-Forlanini, Circonvallazione Gianicolense 83, 00100 Rome, Italy; 3 Departement of Surgery, Azienda USL Toscana Centro, Ospedale San Giovanni di Dio, Via Torregalli 3, 50143 Florence, Italy; ferdinando.delprete@uslcentro.toscana.it; 4 Departement of Surgery, Azienda USL Toscana Centro, Ospedale Santa Maria Annunziata, Via Antella, 58, Ponte a Niccheri, Bagno a Ripoli, 50012 Florence, Italy; giacomo.sani@usltoscana.centro.it; 5 Clinical Laboratory, Department of Immunologia e Allergologia, Azienda USL Toscana Centro, Ospedale San Giovanni di Dio, Via Torregalli 3, 50143 Florence, Italy; mariangela.manfredi@usltoscana.centro.it

**Keywords:** proximal lateral femur fractures, cytokines, bone tissue damage serum markers, biomarkers, interleukins, tissue-sparing surgery, femur neck fractures, frail elderly

## Abstract

**Background/Objectives**: Limited surgical invasiveness is desirable in elderly patients with femur fracture. Serum cytokines have been considered as a possible marker but with inconclusive evidence. The present study aimed to assess the systemic inflammatory response to surgical trauma through the serum levels of several cytokines (IL1β, IL6, IL8, and IL-10), inflammatory markers (c reactive protein—CRP), and muscular damage markers (creatinkinase—CK) at different time intervals in a consecutive series of patients affected by pertrochanteric fractures (PFs) and treated by two different surgical devices (intramedullary nailing (IM) vs. dynamic hip screw plate—DHS). **Methods**: A total of 60 consecutive patients (45 female and 15 male, mean age 85.6 years) with PFs (AO31A1.2-2.2) were randomly assigned to two groups according to the surgical procedure used (IM vs. DHS). Specimens of venous blood were collected 1 h preoperatively and at 24, 48, and 72 h postoperatively. Commercial ELISA kits were used. **Results**: In the adjusted linear mixed model, the serum levels of IL-1β, IL-8, IL-10, CRP, and CK revealed no statistically significant correlation with the type of surgical intervention performed. A significant (*p* < 0.001) correlation was found for IL-6 values in patients undergoing IM, showing higher serum values than patients receiving DHSs in all postoperative blood sample collections. **Conclusions**: The results of this study reveal that the use of DHSs may have less biological impact than IM in frail elderly due to a more limited secretion of IL-6 cytokines deriving from the preservation of the femoral medullary canal, representing a possible guide for the choice of the surgical device.

## 1. Introduction

Pertrochanteric fractures (PFs) are one of the most common fracture patterns in the elderly, with a reported high mortality rate (9%, 19%, and 30% at thirty days, ninety days, and one year, respectively) [1,2]. Although surgical treatment is considered mandatory whenever possible, it represents a further trauma for such frail patients; consequently, minimally invasive methods for fracture reduction and fixation are sought [3,4]. Current evidence in the literature shows that interleukins (ILs) have a role in the inflammatory response to traumas, and they may potentially represent a reliable marker, although highly sensitive but low specific [4,5]. In particular, IL-6 was more deeply assessed in trauma and critically ill patients because of its relatively long detectability in the blood as well as the easy availability of detection kits [6,7]. Moreover, a strong correlation between the magnitude of the inflammatory response and adverse outcomes and IL-6 serum concentrations has been reported in previous studies for traumatized patients [7], confirming that elevated IL-6 values do correlate with adverse outcomes [7,8]. IL-11 was also assessed in the trauma setting, although no correlations with clinical outcomes were detected in study literature [9].

Dynamic hip screws (DHSs) have been considered for decades the gold standard for stabilizing PFs, with preservation of the medullary canal despite a wider soft tissue dissection [10]. On the contrary, intramedullary nailing (IM) has been considered more useful for unstable or reverse pattern fractures, with a more limited soft tissue dissection [11,12] despite medullary canal violation. However, currently, many surgeons adopt both devices independently from the type of fractures, and in experienced hands, both these procedures may be considered minimally invasive [13,14].

In the present study, we assessed the systemic inflammatory response to surgical trauma through the serum levels of several ILs (IL1β, IL6, IL8, and IL-10), inflammatory markers (c reactive protein—CRP), and muscular damage markers (creatinkinase—CK) at different time intervals in the preoperative and in the postoperative period, in a consecutive series of patients affected by PFs and treated with by two different surgical devices (IM vs. DHS).

The primary aim was to ascertain whether ILs are sensitive markers for surgical invasiveness; the secondary aim was to verify whether the value of serum ILs was correlated to the type of surgical device adopted.

## 2. Materials and Methods

### 2.1. Study Design and Patients

This was a prospective randomized study. The study protocol was approved by our institutional ethics committee (Sapienza n°0562/2022). Written informed consent was obtained from all patients according to the Declaration of Helsinki.

Between January 2020 and April 2021, ninety-eight consecutive patients affected by PFs (31 type A subgroups 1.2 and 2.2, according to the AO classification [15]), presenting at the emergency departments of Azienda Ospedaliero-Universitaria Careggi (Florence, Italy) Hospital and San Giovanni di Dio Hospital (Florence, Italy), were initially considered for the present study. A standardized joined study protocol was subscribed, approved, and applied by the two participating structures.

Exclusion criteria were as follows: <70 years; cardiovascular diseases requiring any medical treatment before surgery; steroids or NSAIDs consumption in the last 10 days; active or chronic infection; polytrauma; autoimmune diseases. Sixty-two patients were considered eligible and randomly assigned to two different treatment groups (sealed envelopes): group 1, plate and screw fixation with the DHS^®^ (DePuy-Synthes, Warsaw, IN, USA); group 2, closed reduction and internal fixation by Gamma3^®^ nail (Stryker, Kalamazoo, MI, USA). Two senior skilled trauma surgeons (FDP and RC) performed all of the surgical procedures.

Standard x-rays (AP and lateral views) were performed to classify fractures according to the AO classification: controversies were solved by consensus. Clinical data, trauma to surgical theatre time interval, operating time, incision length, fixation type, intraoperative complications, blood transfusions (BTs), visual analogue scale (VAS), and body mass index (BMI) were registered for each patient. The blood transfusion regimen applied at our institution was hemoglobin (Hb) values < 9 g/dL or hematocrit < 27%.

In the two groups, the interleukin values (serum levels of IL-6, IL-10, IL-8, and IL1-β) were measured at admission time in the surgical ward on the day of fracture and in the first three days post surgery; a 24 h interval between each blood sample analysis was applied. Each patient underwent the surgical procedure within 24 h from the initial admission according to our internal protocol.

### 2.2. Laboratory Examinations

Specimens of venous blood were collected 1 h preoperatively and 24, 48, and 72 h postoperatively, and all blood samples were processed by the same clinical immunology laboratory with the following protocol: after centrifugation at 3000 rpm for 5 min, sera were collected, divided in 3 aliquots for each sample, and stored at −80 °C, until dosages were performed. All samples were analyzed anonymously by the same lab technician. Levels of IL-1β, IL-6, IL8, and IL10 were quantified by commercial ELISA kits (Invitrogen^®^—Thermo Fisher Scientics, Waltam, Massachusets, USA—IL-1β/IL-6/IL8/IL10 Instant ELISA Kit, ThermoFisher-Bender MedSystems GmbH, Vienna, Austria). Healthy donors’ control values were as follows: 8.7 pg/mL for IL-1β, 5.8 pg/mL for IL-6, 114 pg/mL for IL-8, and 9.6 pg/mL for IL-10. CRP was measured by immunophelometry, normal value ≤ 5 mg/L and plasma level of CK were measured via a colorimetric method, with the normal value ≤ 215 UI/L (Cobas 8000 Roche Diagnostics, Rotkreuz, Switzerland).

### 2.3. Surgical Techniques

The traction table was used to perform indirect reduction of the fracture under fluoroscopy control.

### 2.4. DHS—Minimally Invasive Percutaneous Osteosynthesis (MIPO)

Skin incision was performed below the lesser trochanter, for the length necessary to allow the insertion of the guide-wire aiming device. After fascia incision, the vastus lateralis was spread upwards for the entire length of the incision, without passing through the muscle fibers and without affecting the proximal insertion. After femur neck reaming and neck screw placement, the insertion of the plate was carried out by sliding it beneath the vastus lateralis muscle without the use of the guide rod. If necessary, a trochanteric stabilizing plate (TSP) and an anti-rotation screw were applied, always under fluoroscopy control.

### 2.5. Intramedullary Nail

From a short incision on the skin 2 cm above the apex of the greater trochanter, the fibers of the middle gluteus were spread for the insertion of the guide wire under fluoroscopy control. Once the first part of the femur had been drilled, the intramedullary nail was inserted and then the screws or the lag screw were inserted; the distal locking of the nail was then performed, if necessary, with a bicortical screw positioned percutaneously.

### 2.6. Statistical Analysis

A sample size calculation conducted before the study revealed that a minimum sample of 55 patients was necessary for study purposes by assuming a mean difference of 7 pg/mL between the two groups for each assessed IL value (alpha 0.05; beta 0.2; power 0.8). The baseline characteristics of patients of both groups were compared using a *t*-test for continuous variables (e.g., age, BMI, preoperative Hb values), chi-square for 2 × 2 contingency tables (e.g., sex, surgical site), and Fisher’s exact test for contingency tables with numbers of rows/columns greater than 2 (e.g., comorbidities, fracture pattern). For each inflammatory and soft-tissue damage biomarker (IL-1β, IL-6, IL-8, IL-10, CRP, CPK), we computed the difference (delta) of the serum levels of the biomarker between the preoperative (time 0) and 1, 2, and 3 days after surgery. A descriptive analysis of deltas for each biomarker was shown, along with a line chart of the fluctuations within the preoperative evaluation and the first 3 days after the procedure. To investigate the impact of the surgical technique on the delta of inflammation and soft tissue damage biomarkers, we used crude and adjusted linear mixed-effects models with a random slope. We decided to use a random slope linear mixed-effects model instead of linear regression, in order to take into account the by-subject variability of the biomarkers. Crude models were built by including the surgical technique as the dependent variable and the delta of the biomarker under investigation as an independent variable in the statistical model. In the adjusted models, we conditioned for age, the time of measurement of the biomarker, the medical history of diabetes mellitus, hypertension, thyroid disorders, osteoporosis, and cardiomyopathy, and the length of the surgical procedure. The level of significance was set at 0.05 for all tests. R^®^ (version 4.0.0, R development core team, Vienna, Austria) was used for the statistical analysis.

## 3. Results

One patient presented Mallory–Weiss syndrome in the postoperative period and was excluded (group1); one patient from group 2 suffered from an acute myocardial infarction on the second postoperative day and was consequently excluded. Sixty patients were left for the present study. There were 45 female and 15 male patients with a mean age of 85.6 years (range: 79–93). There were no statistically significant differences between the two groups (*p* > 0.05) for the assessed demographical and surgical variables reported in Table 1. Mean values, standard deviations (SDs), and ranges of the assessed variables are reported in Table 2.

**Figure 1:**
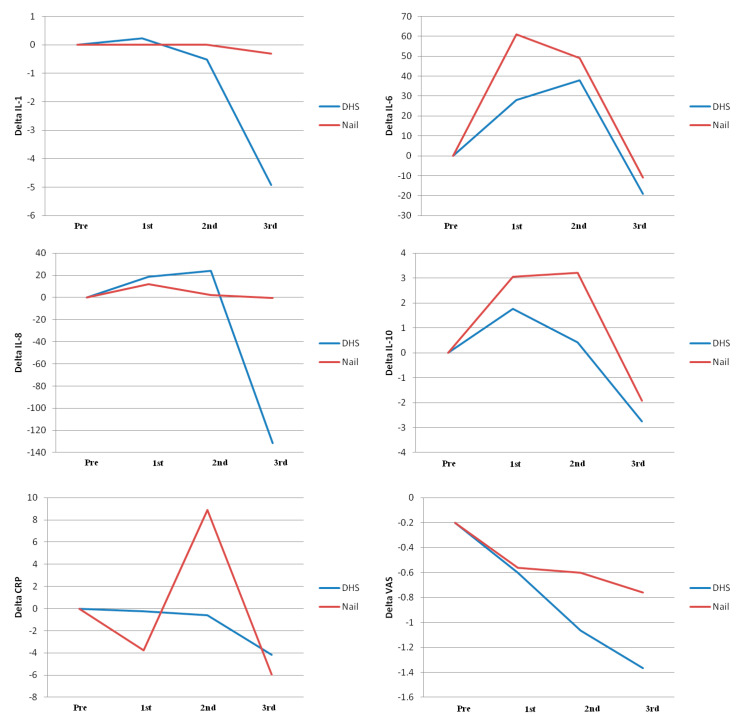
The IL, CRP, CK, and VAS delta mean serum differences trends in the observed time intervals are shown in this figure. DHS: dynamic hip screw.

Gamma3^®^ was used in 30 patients and DHS^®^ in 30 patients. The mean surgical time was 54.2 min (SD 18.3) in group 1 and 56.4 min (SD 19.5) in group 2. No significant differences were found when considering the mean surgical time between group 1 and group 2 (*p* = 0.6). The mean blood transfusion rate was 1.5 (SD, 1.3) in group 1 and 0.8 (SD 1.1) in group 2, with no significant differences between the two groups (*p* = 0.052) (Table 1). IL, CRP, CK, and VAS delta serum differences trends are shown in Figure 1.

**Figure 2:**
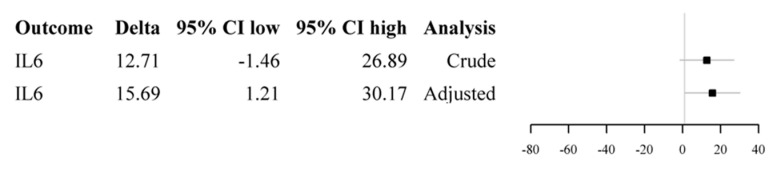
The figure shows the forest plot of the crude (upper lines) and adjusted (lower lines) linear mixed model for the IL-6 delta values. In the adjusted model, a delta value of 15.69 (95%CI, 1.21–3.17) reveals a significant correlation between the variable and the performed surgical technique (cephalomedullary nail vs. dynamic hip screw—DHS).

When considering the crude models of the linear mixed-effect model for the two groups correlating the type of fixation device and the delta (i.e., the absolute difference between the means of the serum biomarkers levels between the preoperative day and the first, second, and third postoperative days) of the assessed biomarkers (IL1β, 6, 8, 10, CRP, and CPK), no significant correlations were found between the two variables (i.e., the surgical technique and the increases in the serum values of the assessed biomarkers). Otherwise, when looking at the adjusted models corrected for age, time, medical history, and length of surgical procedure, a significant correlation (*p* = 0.03) was found only for the level of IL6 (delta 15.6, 95% CI 1.2–30.1) (Figure 2).

## 4. Discussion

The results of our study revealed a statistically significant difference in the adjusted correlation model between the plate (DHS) and the nail group for the postoperative IL-6 serum values, with patients in the nail group reporting higher IL-6 serum values than the plate group, with a mean difference between the groups of 15.7 pg/mL at each time interval. Such data may suggest that patients undergoing nail fixation suffer major surgical trauma probably due to the violation of the femoral medullary canal caused by the proximal reaming and the nail itself, with a higher cytokine release in the immediate postoperative period, independent of age and sex.

The surgical fixation of PFs in frail patients is the gold standard of treatment. However, this should be achieved through the least invasive surgical technique and the faster operative time, due to the potential consequences of the “second hit” on the physiological equilibrium of frail patients. What constitutes a “less invasive and faster” surgical intervention is still a topic of debate and controversy [3,4,16]. Actually, no standard methods for objective quantification of surgical invasiveness are available, although several studies tried to assess the role of cytokines (IL-6, TNFα, IL-8) and biomarkers of tissue trauma (CRP, CK) [17,18,19]. The body’s response to trauma is a complex network of cells activating specific patterns of cytokine secretion [5,19], with a fine equilibrium between pro- and anti-inflammatory cytokine production also influenced by several variables, such as age, gender, medical comorbidities, and the degree of injury suffered [20,21].

On the basis of the current evidence, we hypothesized that ILs (IL-1β, 6, 8, 10) and tissue damage biomarkers (CRP, CK) could be reliable markers for surgical invasiveness in the treatment of PFs, and we verified if any correlation existed between their serum values and the surgical device used to treat PF.

To accomplish such a goal, determining the serum levels of cytokines and serum biomarkers of tissue trauma in a consecutive series of patients affected by PF (“first hit” trauma) was initially performed and at a 24 h interval after the surgical intervention (“second hit”) for the first three days. The 24 h interval was felt to be a reliable time interval to assess serum biomarkers fluctuations, according to previous studies [17,19]. In order to reduce the effect of any possible confounding variable on the serum levels of cytokines and damage biomarkers, rigorous selection criteria of the patients were applied.

According to the results of the present study, the DHS plate may have a biological advantage especially in frail subjects, since the IL-6 values directly related to the magnitude of the systemic inflammatory phase and to the negative consequences of a predominant pro-inflammatory state [7,8,9,22,23,24]. In our cohort, IL-6 values increased in the first postoperative day and then decreased in the following 4 days, with a similar trend in DHS and nail groups; however, absolute values at each examination point differed significantly between the two groups, with patients in the DHS group reporting lower values. This confirmed our secondary hypothesis that IL serum values positively correlate with the surgical device used.

Such data are in line with what was reported in a previous study by Del Prete et al. [18]. As for surgical invasiveness prediction, on the basis of the known relationship between IL values and the degree of trauma severity, we can speculate that intramedullary devices are more invasive than extramedullary devices (plate and screws), and this could turn into a practical point when deciding the most appropriate surgical device in femur fractures, especially in patients for whom pleiotropic effects of IL-6 are expected to foresee adverse clinical outcomes due to concomitant comorbidities. Other studies in the literature, in particular, reported that elevated IL-6 and IL-8 serum values contribute to organic delirium onset in the elderly [25] and predict negative outcomes in this population [26,27]. Finally, recent studies on the effect of IL inhibitors in the COVID-19 pandemic confirmed the importance of cytokine modulation to prevent diffuse inflammatory damages [28,29].

The results of our study seem to be in contrast with those reported by Marino et al. [17], where no difference was assessed between the preoperative (1 h before surgery) and postoperative (24 h after surgery) values of IL-6 in patients receiving nail fixation for a PF: according to this study, the violation of the medullary canal would not generate higher IL-6 serum concentrations than the original fracture. Interestingly, CRP, an IL-6-induced peptide, revealed a significant increase postoperatively in their series.

Wang et al. [26] reported that a significant difference in the serum levels of IL-6 could be detected 7 days after the surgical procedure, with patients receiving nails presenting higher values than subjects receiving DHSs. Due to serum sampling limited to the first three days after surgery in our series, we are not able to speculate about the meaning of these findings, although a decreasing trend in IL-6 serum values was observed in the days following the surgical procedure. Differences in patient selection, in the surgical protocol, and in the timing of sampling adopted may explain such these differences.

No significant differences between the groups in our study were found for other cytokines (IL-1, 8, 10). Differently from previous studies [18], we did not find a steep reduction in serum values of IL-8 after the surgical procedure, so the significance of this pattern remains uncertain. According to recent literature, IL-8 not only represents an inflammatory cytokine but it is also involved in bone metabolism, with an active role in the remodeling phase [29]: consequently, it would be logical to obtain a more stationary serum concentration of this cytokine in the postoperative period as we observed in our series, even if for a limited period. Furthermore, the values of CPK and CRP revealed no significant differences between the two groups; these results seem to be supported also by the study of Hong et al. [19].

Although not statistically significant, some trends in the variations of the delta values for the assessed biomarkers at the different dosage times could be detected. A general trend of progressive reduction was found for most of the assessed variables, with a steep reduction between the second and third postoperative days, particularly evident for CPK. Interestingly, the IL-1β values in the nail group were quite stable over time; on the contrary, a progressive reduction with a certain drop in the third postoperative day was observed for the IL-1β serum values in the plate group. IL-8 showed a more linear trend in all patients, with a more discrete reduction of the values over time.

This study has several limitations. First, the role of ILs in the quantification of the surgical invasiveness of PF fixation is actually limited by the lack of standardized methods (timing, number of blood samples necessary, etc.) and the extreme variability in the humoral response due to multiple confounding variables. Second, the limited sample and the multicentric nature of the study restricted the scope of study, despite a shared surgical protocol.

This study has also several strengths, such as the rigorous patient inclusion and exclusion criteria and the meticulous study design that prevented us from inclusion biases and data misinterpretation.

## 5. Conclusions

The results of the present study reveal that far from biomechanical perspectives, the use of a plate seems to have a biological advantage over intramedullary nailing due to the more limited secretion of ILs, in particular IL-6, deriving from the preservation of the femoral medullary canal. Such aspects could be considered in the choice of surgical device in the frail elderly. However, more studies with larger cohorts of patients are needed to support such findings.

## Figures and Tables

**Table 1:** Patient data of DHS and nail groups.

Variables (Mean, SD)	DHS (n = 30)	Nail (n = 30)	*p* Value
**Age**	86 (6.3)	81.9 (20.5)	0.3
**Gender**			1
Male	7	8	
Female	23	22	
**Surgical side**			0.89
Left	13	14	
Right	17	16	
**Hgb (pre operative)**	11.2 (1.3)	11.8 (1.8)	0.13
**Type of fracture**			0.75
A1	9	11	
A2	21	19	
**Number of transfusions**	1.5 (1.3)	0.8 (1.1)	0.052
**Surgical time (min)**	54.2 (18.3)	56.4 (19.5)	0.66
**BMI**	23.3 (3.4)	24.7 (4.3)	0.14
**Mean number of comorbidities**	2.4 (0.97)	2.3 (1.3)	0.7

Hgb: hemoglobin; BMI: body mass index.

**Table 2:** Means, standard deviations, and ranges of interleukins (ILs), CK, and CRP at different time intervals.

Variables	DHS (n = 30); Mean (SD), Range	Nail (n = 30); Mean (SD), Range	*p* Value
IL-1 (pg/mL)			0.6
pre	14.8 (13.3); 7.8–42.6	6.8 (1.2); 5.6–7.9	
1st postoperative day	16.6 (17.2); 7.8–53.3	7.1 (2.1); 5–9.2	
2nd postoperative day	13.7 (10.7); 7.8–34.8	7.8 (0.6); 7.2–8.3	
3th postoperative day	11.6 (6.9); 7.8–25.5	8.8 (1.1); 7.7–9.9	
IL-6 (pg/mL)			0.03 *
pre	39 (13.5); 18.6–62.8	19.6 (7.6); 13–30	
1st postoperative day	49.9 (51.9); 4.1–159.4	67.2 (52.8); 18.5–138	
2nd postoperative day	75.2 (56.6); 26.2–173.3	47.8 (41.5); 5.3–108.3	
3th postoperative day	42.4 (40.1); 10.2–129.1	13.6 (5.4); 8–21	
IL-8 (pg/mL)			0.5
pre	69.8 (96.6); 32–389	33.8 (3.6); 32–39.3	
1st postoperative day	192.2 (416);32–1136	101.1 (124); 32–286	
2nd postoperative day	32.4 (1.2); 32–35	41.6 (19.3); 32–71	
3th postoperative day	32.1 (0.37); 32–33	32 (0); 0	
IL-10 (pg/mL)			0.8
pre	5.4 (3.04); 3.1–9.9	20.8 (35.5); 3.1–74.1	
1st postoperative day	7.7 (4.5); 3.1–13.2	36.2 (54.4); 3.1–113.1	
2nd postoperative day	6.07 (3.7); 3.1–11.8	40.6 (75); 3.1–153.1	
3th postoperative day	4.07 (1.42); 3.1–6.4	6 (4.3); 3.1–12.3	
CK (UI/L)			0.7
pre	196.5 (164); 33–456	162 (56.2); 93–218	
1st postoperative day	328.7 (205); 65–623	336 (89.7); 218–424	
2nd postoperative day	403.1 (290.7); 101–875	333.2 (112); 211–483	
3th postoperative day	180.5 (101.4); 48–328	136 (27); 102–165	
CRP (mg/mL)			0.9
pre	7.8 (4.6); 1.5–13.1	6.8 (2.6); 3.2–9.6	
1st postoperative day	7.2 (4.3); 1.9–13.8	7.2 (2.2); 4.5–9.4	
2nd postoperative day	11.4 (4.5); 4.9–16.4	9.2 (2.6); 6.1–11.7	
3th postoperative day	6.6 (4.5); 1.4–14.1	6 (1.95); 3.9–8.4	
VAS			0.5
pre	2.5 (0.8); 1–4	2.9 (1.6); 0–5	
1st postoperative day	2.9 (1.2); 2–6	2.9 (1.6); 0–6	
2nd postoperative day	2.2 (0.9); 0–4	2.7 (1.4); 0–6	
3th postoperative day	1.8 (0.7); 0–3	2.3 (1.5); 0–4	
IL-1 (pg/mL)			0.6
pre	14.8 (13.3); 7.8–42.6	6.8 (1.2); 5.6–7.9	
1st postoperative day	16.6 (17.2); 7.8–53.3	7.1 (2.1); 5–9.2	
2nd postoperative day	13.7 (10.7); 7.8–34.8	7.8 (0.6); 7.2–8.3	
3th postoperative day	11.6 (6.9); 7.8–25.5	8.8 (1.1); 7.7–9.9	
IL-6 (pg/mL)			0.03 *
pre	39 (13.5); 18.6–62.8	19.6 (7.6); 13–30	
1st postoperative day	49.9 (51.9); 4.1–159.4	67.2 (52.8); 18.5–138	
2nd postoperative day	75.2 (56.6); 26.2–173.3	47.8 (41.5); 5.3–108.3	
3th postoperative day	42.4 (40.1); 10.2–129.1	13.6 (5.4); 8–21	
IL-8 (pg/mL)			0.5
pre	69.8 (96.6); 32–389	33.8 (3.6); 32–39.3	
1st postoperative day	192.2 (416); 32–1136	101.1 (124); 32–286	
2nd postoperative day	32.4 (1.2); 32–35	41.6 (19.3); 32–71	
3th postoperative day	32.1 (0.37); 32–33	32 (0); 0	
IL-10 (pg/mL)			0.8
pre	5.4 (3.04); 3.1–9.9	20.8 (35.5); 3.1–74.1	
1st postoperative day	7.7 (4.5); 3.1–13.2	36.2 (54.4); 3.1–113.1	
2nd postoperative day	6.07 (3.7); 3.1–11.8	40.6 (75); 3.1–153.1	
3th postoperative day	4.07 (1.42); 3.1–6.4	6 (4.3); 3.1–12.3	
CK (UI/L)			0.7
pre	196.5 (164); 33–456	162 (56.2); 93–218	
1st postoperative day	328.7 (205); 65–623	336 (89.7); 218–424	
2nd postoperative day	403.1 (290.7); 101–875	333.2 (112); 211–483	
3th postoperative day	180.5 (101.4); 48–328	136 (27); 102–165	
CRP (mg/mL)			0.9
pre	7.8 (4.6); 1.5–13.1	6.8 (2.6); 3.2–9.6	
1st postoperative day	7.2 (4.3); 1.9–13.8	7.2 (2.2); 4.5–9.4	
2nd postoperative day	11.4 (4.5); 4.9–16.4	9.2 (2.6); 6.1–11.7	
3th postoperative day	6.6 (4.5); 1.4–14.1	6 (1.95); 3.9–8.4	
VAS			0.5
pre	2.5 (0.8); 1–4	2.9 (1.6); 0–5	
1st postoperative day	2.9 (1.2); 2–6	2.9 (1.6); 0–6	
2nd postoperative day	2.2 (0.9); 0–4	2.7 (1.4); 0–6	
3th postoperative day	1.8 (0.7); 0–3	2.3 (1.5); 0–4	

IL: interleukin; CK: creatine kinase; CRP: C reactive protein; VAS: visual analogic scale; *: statistical significance.

## Data Availability

The original contributions presented in this study are included in the article. Further inquiries can be directed to the corresponding author.
